# Potential Public Health and Economic Impact of the Next-Generation COVID-19 Vaccine mRNA-1283 in The Netherlands

**DOI:** 10.3390/vaccines14040364

**Published:** 2026-04-20

**Authors:** Simon van der Pol, Ekkehard Beck, Tjalke Westra, Maarten Postma, Cornelis Boersma

**Affiliations:** 1 Department of Health Sciences, University Medical Center Groningen, University of Groningen, 9700RB Groningen, The Netherlands; 2 Health-Ecore, 3704HE Zeist, The Netherlands; 3 Moderna, Inc., Cambridge, MA 02142, USA; 4 Moderna Netherlands, 1082MC Amsterdam, The Netherlands; 5 Department of Economics, Econometrics and Finance, University of Groningen, 9747AE Groningen, The Netherlands; 6 Department of Management Sciences, Open University, 6401DL Heerlen, The Netherlands

**Keywords:** cost-effectiveness, COVID-19, economic model, Netherlands, public health, SARS-CoV-2, vaccination

## Abstract

Background: COVID-19 remains a substantial public health challenge in the Netherlands. Next-generation COVID-19 vaccine, mRNA-1283, is approved in the European Union, with potential for higher relative vaccine efficacy compared with originally licensed COVID-19 vaccines. Methods: The potential public health and economic impact of mRNA-1283 in adults ≥ 60 years and high-risk adults aged 18–59 years was modeled versus no vaccination and originally licensed mRNA-1273 and BNT162b2, adapting a published static Markov model with a 1-year time horizon. COVID-19 burden reflected two full post-pandemic seasons. Vaccine efficacy versus mRNA-1273 was based on pivotal phase 3 NextCOVE trial data; efficacy versus BNT162b2 was derived from an indirect treatment comparison. The economically justifiable price (EJP) of mRNA-1283 versus no vaccination and price premiums over existing vaccines were determined at a willingness-to-pay threshold of €50,000/quality-adjusted life-year (QALY) gained. Results: Without COVID-19 vaccination, an estimated 460,000 infections, 23,800 hospitalizations, and 5300 deaths would occur. With current coverage, mRNA-1283 was estimated to prevent 68,000 infections, 5400 hospitalizations, and 1200 deaths, saving 9667 QALYs and over €66.5 million in treatment costs. The EJP was €238 versus no vaccination. Compared with mRNA-1273 and BNT162b2, mRNA-1283 was estimated to prevent additional burden (e.g., 1309 and 1679 hospitalizations, respectively) and was cost-effective at an incremental EJP of €62 versus mRNA-1273 and €80 versus BNT162b2. Conclusions: The results support continued COVID-19 vaccination to mitigate the ongoing health and societal burden of SARS-CoV-2 in the Netherlands. The comparative analyses indicate that mRNA-1283 may be associated with substantial health benefits over originally licensed mRNA vaccines; consequently, its use may further improve health outcomes and economic efficiency within COVID-19 vaccination programs.

## 1. Introduction

The COVID-19 pandemic has had a profound and sustained impact on population health and patient well-being, placing unprecedented pressure on healthcare systems worldwide. Since the introduction of mRNA COVID-19 vaccines, substantial reductions in severe disease, hospitalizations, and mortality have been observed. These vaccines were developed to provide strong protection against COVID-19, and their high effectiveness has been consistently demonstrated in both clinical trials [1,2,3] and real-world studies [4,5], including evidence from the Netherlands showing robust protection against severe outcomes [6].

In recent years, the Dutch Health Council has narrowed its recommendations for COVID-19 vaccination [7]. Current guidelines recommend vaccination primarily for individuals aged 18 years and older at increased medical risk, adults aged ≥ 60 years, adults aged ≥ 50 years who are also eligible for seasonal influenza vaccination, and healthcare workers [7]. This policy shift reflects a declining annual burden of disease, with reductions in reported cases, hospital admissions, and COVID-19-related deaths [8].

However, COVID-19 continues to pose a substantial public health challenge in the Netherlands. Hospitalizations and mortality attributable to COVID-19 persist, in particular, among those most vulnerable to severe COVID-19 disease, i.e., people with underlying medical conditions and older adults ≥ 60 years of age [7]. Modeling studies, as well as observational data, suggest that in the absence of vaccination, these numbers would be substantially higher [9]. The decreasing trend in vaccination coverage, from around 47% in 2024 to around 42% in 2025 [10], is also a cause for concern. Moreover, COVID-19 has multiple peaks throughout the year, and the largest peak coincides with influenza and respiratory syncytial virus (RSV) circulation during the winter months [7,11]. This temporal overlap increases pressure on healthcare capacity, underscoring the continued importance of effective preventive strategies [8].

A next-generation COVID-19 vaccine, mRNA-1283 (mNEXSPIKE, Moderna, Cambridge, MA, USA), has recently been approved in the European Union [12]. It was developed to focus the immune responses to increase protection against COVID-19 at a lower mRNA dose of 10 μg (e.g., 1/5th of the dose of mRNA-1273 (Spikevax, Moderna [13]), and its innovative design allows for inclusion in respiratory combination vaccines in future further developments. In pivotal phase 3 randomized clinical trials, higher immunogenicity and a higher relative vaccine efficacy (rVE) point estimate were observed with mRNA-1283 versus mRNA-1273, with the highest differences observed in adults ≥ 65 years and those with underlying medical conditions [3,14]. Observational studies and GRADE meta-analyses have shown that mRNA-1273 was more effective than BNT162b2 (Comirnaty, Pfizer-BioNTech, Mainz, Germany and New York, NY, USA) in the real-world setting [15,16,17,18,19]. Building on this evidence, and an indirect treatment comparison suggesting mRNA-1283 may be more effective than BNT162b2 [20], it is essential to understand the potential added clinical and economic value of mRNA-1283 over originally licensed mRNA COVID-19 vaccines.

The objectives of this study were to assess the potential public health and economic impact of mRNA-1283 compared with no COVID-19 vaccination, considering the current endemic and seasonal COVID-19 context. Furthermore, the impact of mRNA-1283 vaccination was compared with mRNA-1273 and BNT162b2.

## 2. Materials and Methods

The public health impact and economically justifiable price (EJP) of the next-generation mRNA-1283 vaccine versus no vaccination, mRNA-1273, and BNT162b2 were estimated in the Netherlands with three different scenarios based on the post-pandemic epidemiology. A previously published static Markov decision tree model with monthly cycles comparing mRNA-1273 to no vaccination and to BNT162b2 vaccination over a 1-year time horizon in the Netherlands [9] was adapted and updated for this analysis.

### 2.1. Target Population

The base case target vaccination population included all adults aged ≥ 60 years and high-risk adults aged 18–59 years, defined as individuals at a high medical risk aged 18–49, and the influenza target group aged 50–59 years, according to season 2025/2026 Dutch COVID-19 vaccination recommendations [7].

### 2.2. Model Structure and Inputs

**Figure A1:**
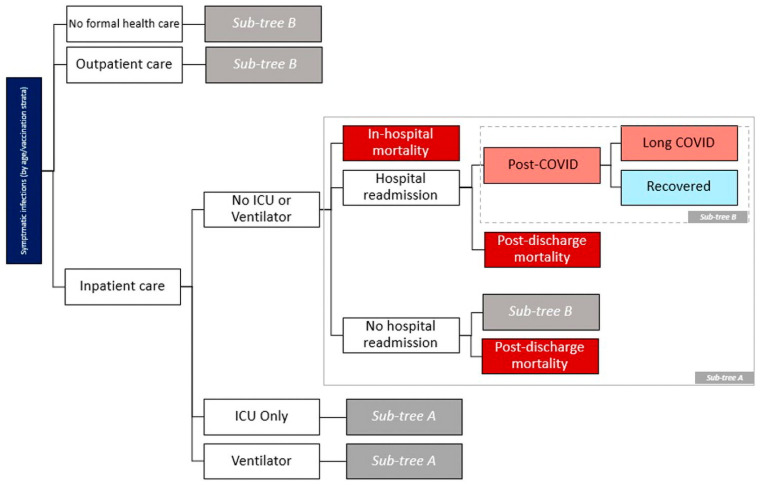
Decision tree consequences model (based on [21]). ICU: intensive care unit.

The model structure and assumptions were previously described for economic analyses in the Netherlands (Zeevat et al. 2025 [9]), the United States (US) [21], and Canada [22] (Figure A1). In summary, individuals had a monthly risk of COVID-19 infection based on Dutch age-specific incidence data. Following infection, the risk of health outcomes was determined by the decision tree. Every month, individuals could receive a COVID-19 vaccine, based on age-specific coverage rates, which reduced the risk of infection and hospitalization following infection, according to each vaccine’s efficacy (Table 1). Following Zeevat et al. 2025 [9], a static model was considered appropriate, though conservative compared with a dynamic modeling approach, due to the restriction of COVID-19 vaccination to high-risk populations in the Netherlands, a short model time horizon, and other endemic setting cost-effectiveness modeling approaches in the US [23] and UK [24].

The model inputs and assumptions (Table A1) were previously described in Zeevat et al. 2025 [9], with updates to input parameters summarized below. All other input parameters follow Zeevat et al. 2025 [9]. Cost estimates, including productivity losses, from Zeevat et al. 2025 were inflated from 2023 to 2025 using the Dutch consumer price index [25].

The societal perspective was considered for the analyses, as in Zeevat et al. 2025 [9] and aligned with Dutch guidelines [26].

### 2.3. Model Input Parameter Updates

As COVID-19 incidence is evolving and remains subject to seasonal fluctuations, the base case was intended to represent a plausible endemic/seasonal steady-state, as is often done for influenza modeling [27], rather than to forecast future epidemic resurgence. Thus, the base case risk of infection assumed the average incidence of symptomatic COVID-19 infections in the 2023/2024 and 2024/2025 seasons in the Netherlands. Specifically, the 2023/2024 season was used to represent a relatively high incidence season and was based on the incidence used in Zeevat et al. 2025 [9], whereas the 2024/2025 season was estimated at 40% of 2023/2024 based on the recently published Netherlands infectious disease report of 2024 [8]. The resulting 0.7 scaling factor, therefore, reflects the mean of a recent higher and lower post-pandemic season, rather than a subjective adjustment. Because future COVID-19 incidence remains unclear and resurgence cannot be excluded, separate low and high incidence scenarios were explored in sensitivity analyses.

The vaccine coverage rate (VCR) for the Dutch campaign, running from September to December, was updated using 2025 observed values [10].

A Dutch observational study found that the risk of developing post-COVID-19 condition has declined during post-pandemic years [28]. However, substantial uncertainty remains regarding the current incidence, duration, and severity of post-COVID-19 condition, as well as heterogeneity in its definition and measurement across studies. Given this uncertainty, the post-COVID-19 condition was not included in the base case analysis, in order to avoid introducing structural uncertainty and potential overestimation of vaccine benefits. Thus, contrary to Zeevat et al. 2025 [9], the post-COVID condition was not considered in the base case analysis as part of the infection consequences tree. To ensure transparency, however, the post-COVID-19 condition was considered in a scenario analysis based on the parameters assumed in Zeevat et al. 2025 [9] due to the substantial level of evidence on the risk of onset of post-COVID-19 condition in people infected with COVID-19, especially those most vulnerable to severe COVID-19 disease. In addition, there is an emerging level of evidence that COVID-19, like seasonal influenza and other respiratory diseases, can trigger and exacerbate chronic conditions following a COVID-19 infection [29,30].

### 2.4. Vaccine Characteristics

Vaccine effectiveness (VE) inputs against COVID-19 infection and hospitalization were based on the estimates assumed in a cost-effectiveness analysis of mRNA-1283 in the US for 2025/2026 [21]. In short, the absolute VE of mRNA-1283 was estimated considering a risk-ratio-based approach, applying the rVE of mRNA-1283 versus mRNA-1273 (derived from the pivotal phase 3 NextCOVE randomized clinical trial [3] conducted during 2023–2024 using the bivalent original and Omicron BA4/5 strain containing versions of both vaccines) to the VE of mRNA-1273. The latter was derived from a large real-world evidence study of mRNA-1273 VE (targeting the KP.2 variant) against COVID-19-related hospitalizations and medically attended COVID-19 (a proxy for infections) [31] (Table 1). The rVE of mRNA-1283 versus BNT162b2 was based on findings of an indirect treatment comparison (ITC) [20], leading to absolute BNT162b2 VE estimates (Table 1). An ITC (Bucher method) using the NextCOVE trial data and real-world evidence comparing mRNA-1273 with BNT162b2 estimated higher efficacy with mRNA-1283 than BNT162b2, i.e., estimated rVE against symptomatic infection was 15.3% (95% CI 4.7–24.8%) in all adults and 22.8% (95% CI 4.7–24.8%) in those aged ≥ 65 years. Table 1 first presents the mRNA-1273 VE and the rVE of mRNA-1283 versus mRNA-1273 used to estimate the absolute VE of mRNA, and then presents the absolute mRNA-1283 VE and the rVE of mRNA-1283 versus BNT162b2 used to estimate the BNT162b2 VE.

In the base case, the rVE against hospitalization was based on an FDA-defined severe COVID-19 endpoint of the NextCOVE trial [3], assumed as a proxy for rVE against hospitalization [21]. The same monthly VE waning rates were assumed for all vaccines (4.75% against infection [32] and 2.46% against hospitalization [33]). Given that the rVE of mRNA-1283 versus mRNA-1273 was observed over a median follow-up of 8 months, these estimates are likely to reflect any potential differences in waning. Further details of the estimation of VE inputs for mRNA-1283, mRNA-1273, and BNT162b2 are presented in Fust et al. 2026 [21].

Frequencies of reactogenicity grade 3 events for mRNA-1283 and mRNA-1273 were based on the NextCOVE trial data, and grade 3 events were assumed to be the same for mRNA-1273 and BNT162b2 [22]. Rates of myocarditis/pericarditis (0.0008%) associated with all mRNA COVID-19 vaccines were assumed for high-risk adults aged 18–60 years, following estimates provided in the US FDA label [34].

Updates were made to the utility losses associated with some infection-related health states and with adverse events [22].

### 2.5. Vaccine Unit Costs

COVID-19 vaccine procurement in the Netherlands is conducted through European tenders and commonly includes confidential discounts; thus, net vaccine prices are not publicly disclosed. Consequently, for the economic analysis, the EJP was estimated at a willingness-to-pay (WTP) threshold of €50,000 per QALY gained, based on the proposed WTP threshold in 2024 for public health interventions, including vaccines [9]. In the absence of transparent price data, conventional cost-effectiveness analyses based on fixed vaccine prices would require strong assumptions and may therefore be less appropriate in this context.

### 2.6. Sensitivity and Scenario Analyses

The robustness of the results was assessed in sensitivity and scenario analyses. In particular, the impact of specific parameter assumptions and modeling settings was assessed on the EJP at a WTP of €50,000/QALY. The VE of mRNA-1283 against infection and hospitalization was varied using the upper and lower bounds of the 95% confidence interval; the rVE against hospitalization was varied by applying the same rVE for mRNA-1283 versus mRNA-1273 for infection to hospitalization. The VCR was also varied by ±20%, and further scenario analyses explored restricting vaccination target populations to all adults aged ≥ 75 years only (*n* = 1,759,000), and expanding to all 50–59-year-olds (*n* = 2,465,000), instead of just high-risk 50–59-year-olds (*n* = 298,000) [35,36]. Lastly, a scenario analysis assessed the burden of the post-COVID-19 condition and the impact of vaccination. As in Zeevat et al. 2025 [9], all COVID-19 survivors were assumed to experience, following the acute COVID-19 infection, healthcare resource use and utility loss, while those with post-COVID-19 condition were assumed to experience productivity losses, to avoid double-counting, and due to a lack of data.

Additionally, deterministic sensitivity analyses (DSA) were performed to assess the impact of varying inputs on the ICER of mRNA-1283 vaccination versus no vaccination. Following Zeevat et al. 2022 [37], the base case EJP at a WTP of €50,000/QALY was considered as the vaccine price for this DSA, and was varied along with other input parameters by ±20% (Zeevat et al. 2025 [9]). The DSA results are presented in a Tornado diagram.

Probabilistic sensitivity analyses (PSA) with *n* = 1000 simulations were conducted to assess the impact of parameter uncertainty on incremental EJPs of mRNA-1283 versus mRNA-1273 and mRNA-1283 versus BNT162b2 at a WTP threshold of €50,000/QALY following the approaches taken in Zeevat et al. 2025 [9] and Fust et al. 2025 [22] (see Appendix A for details on parameter input estimates). Table A2 and Table A3 specify each parameter considered in the PSA, the point estimate, the distribution assumed, and the standard error/deviation. The PSA results are presented, showing the likelihood of varying incremental EJPs being cost-effective at a WTP threshold of €50,000/QALY.

## 3. Results

### 3.1. Base Case Analysis: mRNA-1283 Versus No Vaccination

With no COVID-19 vaccination in the base case, among the 14.8 M adults in the Netherlands, there were an estimated 460,000 symptomatic infections, 24,000 hospitalizations, and 5500 deaths from COVID-19.

In the base case, mRNA-1283 vaccination of adults aged ≥ 60 years and of high-risk adults aged 50–59 years (around 2.1 million individuals vaccinated) was estimated to produce important public health gains. Around 68,000 symptomatic infections, 5400 hospitalizations, and 1200 deaths from COVID-19 were estimated to be prevented, with treatment cost savings exceeding €66.5 million and with 9667 QALYs gained (Table 2). The public health impact was estimated to be between 3000 and 7700 hospitalizations prevented, considering a lower or higher COVID-19 incidence (Table 2).

In the base case, the estimated number needed to vaccinate (NNV) with mRNA-1283 was 31 to prevent a symptomatic infection, 396 for a COVID-19 hospitalization, and 1756 for a COVID-19 death.

Given a base case WTP threshold of €50,000/QALY gained, mRNA-1283 was cost-effective at an EJP of €237.93 compared with no vaccination. When considering a WTP of €20,000/QALY, the average EJP of mRNA-1283 across all three incidence scenarios was €101.57.

#### Additional Scenario Analyses and DSA Results

Varying the VE of mRNA-1283 against infection and hospitalization, using the lower bounds and upper bounds of the 95% confidence intervals, resulted in 3754–6693 hospitalizations prevented and 846–1508 deaths prevented, with an EJP of €160.70 (using lower bounds) to €301.05 (using upper bounds) (Table 3). Assuming the same rVE of mRNA-1283 against infection as for hospitalization resulted in fewer prevented hospitalizations (4532) and deaths (1021), and a lower EJP (€199.27) versus the base case. Varying the base case VCR by ±20% resulted in 4300–6451 hospitalizations prevented and 969–1453 deaths prevented, but did not impact the EJP, as a static health economic model was applied. Expanding the target population to all 50–59-year-olds prevented slightly more hospitalizations (5481) and deaths (1219) than in the base case and lowered the EJP to €186.54 (at WTP of €50,000/QALY). By contrast, reducing the target population to only adults ≥ 75 years reduced the number of prevented hospitalizations (2779) and deaths (628) considerably, and increased the EJP to €371.03 compared with the base case. The scenario assessing the impact of mRNA-1283 vaccination on post-COVID-19 condition found that mRNA-1283 would prevent an estimated 2265 incident post-COVID-19 condition cases, at an EJP of €241.96. This represents an increase in estimated economic value and QALY gains compared with the base case analysis.

**Figure 1:**
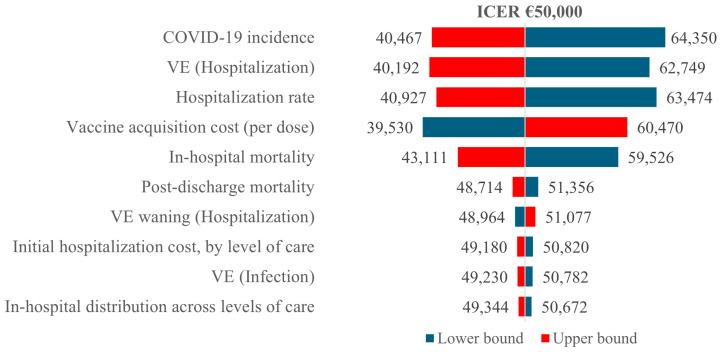
DSA results for mRNA-1283 versus no vaccination. ICER: incremental cost-effectiveness ratio; VE: vaccine efficacy.

Figure 1 shows the ten parameters with the greatest impact on the incremental cost-effectiveness ratio (ICER) of mRNA-1283 versus no vaccination (at an EJP based on WTP of €50,000/QALY). The most important parameters affecting cost-effectiveness were COVID-19 incidence (a higher incidence led to a lower ICER), VE against hospitalization (a higher VE reduced the ICER), and the hospitalization rate given infection (a higher rate of hospitalization led to a more cost-effective vaccination program).

### 3.2. Analysis: mRNA-1283 Versus mRNA-1273

Vaccination with mRNA-1283 prevented more symptomatic infections (8931), hospitalizations (1309), and COVID-19 deaths (295) than mRNA-1273, resulting in treatment cost savings (€14,743,901) and QALY gains (2323). As expected, more vaccination gains were predicted in scenarios with a higher COVID-19 incidence and higher mRNA-1283 versus mRNA-1273 rVE (Table 4).

Using the base case incidence and rVE, mRNA-1283 was cost-effective at an incremental EJP of €61.53 over mRNA-1273 (WTP €50,000/QALY) and €28.77 (WTP €20,000/QALY). These values increased as vaccination benefits increased in the higher incidence and high rVE scenarios, and decreased in the lower incidence scenario, with no incremental EJP estimated in the low rVE scenario (Table 4).

In the base case, the incremental NNV, i.e., the additional number of persons to be vaccinated with mRNA-1273 versus mRNA-1283 to prevent one COVID-19 hospitalization, was 127.

**Figure 2:**
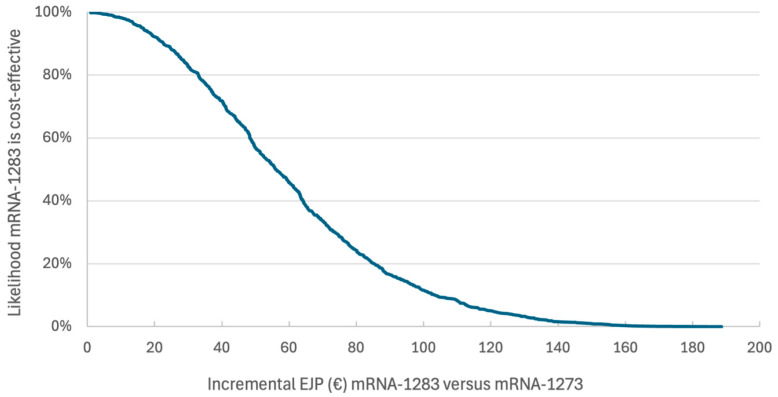
Cost-effectiveness acceptability curve for mRNA-1283 versus mRNA-1273.

With an incremental EJP of €61.53, the probability of mRNA-1283 versus mRNA-1273 being cost-effective at a WTP of €50,000/QALY was 0.44 (Figure 2).

### 3.3. Analysis: mRNA-1283 Versus BNT162b2

Vaccination with mRNA-1283 prevented more symptomatic infections (16,499), hospitalizations (1679), and COVID-19 deaths (378) than BNT162b2, resulting in treatment cost savings (€19,827,071) and QALY gains (3005). Again, more vaccination gains were predicted in scenarios with a higher COVID-19 incidence and higher rVE (Table 5).

Using the base case incidence and rVE, mRNA-1283 was cost-effective at an incremental EJP of €79.96 over BNT162b2 (WTP €50,000/QALY) and €37.57 (WTP €20,000/QALY). These values increased as vaccination benefits increased in the higher incidence and higher mRNA-1283 versus BNT162b2 rVE scenarios, and decreased in the lower incidence and low rVE scenarios, although they remained positive (Table 5).

In the base case, the incremental NNV, i.e., the additional number of persons to be vaccinated with BNT162b2 versus mRNA-1283 to prevent one COVID-19 hospitalization, was 180.

**Figure 3:**
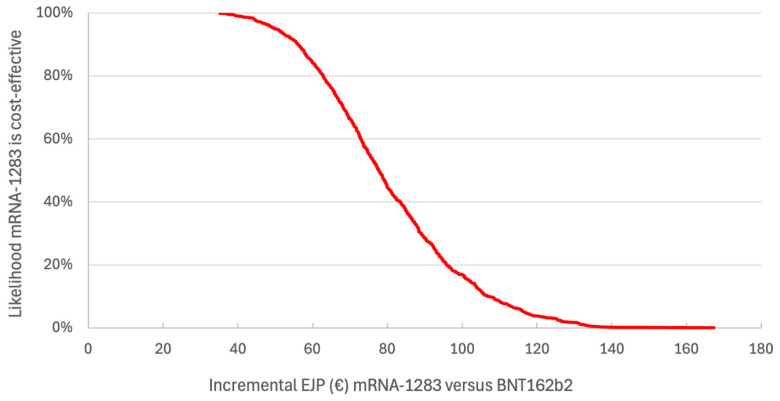
Cost-effectiveness acceptability curve for mRNA-1283 versus BNT162b2.

With an incremental EJP of €79.96, the probability of mRNA-1283 versus BNT162b2 being cost-effective at a WTP of €50,000/QALY was 0.45 (Figure 3).

## 4. Discussion

In the Netherlands, the burden due to COVID-19 remains substantial, especially in those most vulnerable to severe COVID-19 disease. Our results estimated that without vaccination, 460,000 (260,000–660,000) individuals would develop symptomatic COVID-19, leading to 24,000 (13,000–34,000) hospitalizations and 5400 (3000–8000) deaths. Considering the current vaccination coverage and recommended target population, a potential vaccination campaign with mRNA-1283 could prevent 68,000 (39,000–97,000) symptomatic cases, 5400 (3000–8000) hospitalizations, and 1200 (700–1700) deaths compared with no vaccination. These health gains were estimated to lead to 9667 QALYs gained, as well as €66.5 million in cost savings. mRNA-1283 vaccination was estimated to be cost-effective at an EJP of €238 (WTP of €50,000/QALY) and of €102 (WTP of €20,000/QALY).

These model-based estimates, as well as reported surveillance data, suggest that despite widespread population immunity, COVID-19 continues to place a substantial burden on individuals and public health in the Netherlands under current incidence levels. Ongoing transmission results in a large number of symptomatic infections, of which approximately 30% require care from a general practitioner, which can contribute to sustained pressure on primary care services [38]. Importantly, COVID-19 now circulates concurrently with other major respiratory pathogens, including RSV and influenza. The overlapping seasonality of these infections creates a risk of simultaneous epidemic peaks, increasing the likelihood of a so-called “triple endemic” scenario, and increasing the strain on both primary and secondary healthcare capacity. Assuming a COVID-19 hospital length of stay of 8.43 days [39], vaccination with mRNA-1283 could potentially prevent 45,000 hospital bed-days and approximately 17,000 general practitioner visits. Beyond direct healthcare utilization, COVID-19 also imposes a considerable societal burden through productivity losses due to absenteeism and reduced work capacity. Taken together, these factors underline that the current COVID-19 vaccination program remains highly relevant from a public health perspective and from a broader societal standpoint. Maintaining protection against COVID-19 in the working-age population is essential to safeguard workforce availability and ensure the continuity of critical sectors such as healthcare, education, and other essential public services.

Based on pivotal clinical trial data and findings of an indirect treatment comparison, our results estimated that mRNA1283 could provide greater public health benefits than currently available COVID-19 mRNA vaccines, especially in those most vulnerable to severe COVID-19 disease. Compared with mRNA-1273, vaccination with next-generation mRNA-1283 in the base case was estimated to prevent 8900 additional symptomatic COVID-19 infections, 1300 additional hospitalizations, and 295 additional deaths, leading to higher healthcare cost-savings and QALY gains. Even greater health gains and cost-savings were estimated for the comparison of mRNA-1283 versus BNT162b2, the currently used vaccine in the Netherlands [9], in the base case (i.e., preventing around 16,500 additional symptomatic COVID-19 infections, 1700 additional hospitalizations, and 380 additional deaths). Potential vaccination with mRNA-1283 was cost-effective versus mRNA-1273 at an incremental EJP of €62, and versus BNT162b2 at an incremental EJP of €80, considering a WTP of €50,000/QALY for both comparisons. Scenario analyses addressing uncertainty in COVID-19 incidence and the rVE of mRNA-1283 versus mRNA-1273 and mRNA-1283 versus BNT16b2 showed the incremental EJP ranging from 104.65 EUR to 0 EUR and 142.67 EUR to 3.37 EUR when considering a €50,000/QALY WTP threshold. Overall, these findings are in line with other public health and economic evaluations comparing mRNA-1283 vaccination with no vaccination and with existing COVID-19 vaccinations in Canada [22] and the US [21], as well as the previous Dutch evaluation showing the vaccination gains of mRNA-1273 over BNT162b2 [9].

This analysis estimated an EJP of €238 for COVID-19 vaccination. While vaccine prices in the Netherlands are confidential due to tender-based procurement, publicly available information from other European settings provides a useful contextual benchmark. For example, reported list prices for COVID-19 vaccines in countries such as Germany suggest price levels that are substantially below the EJP estimated in this analysis. Although list prices may not reflect actual tender prices, and cross-country comparisons should be interpreted with caution, this observation supports the robustness of our findings. Specifically, it indicates that COVID-19 vaccination is likely to remain cost-effective under a broad range of plausible pricing scenarios. Compared with other preventive interventions for respiratory infectious diseases, such as influenza and RSV vaccination, the COVID-19 EJP seems to be notably higher [9,40,41]. This difference can largely be explained by the substantially higher burden of disease associated with COVID-19 [42,43]. Even in the post-pandemic period, COVID-19 continues to result in a structurally higher number of hospitalizations and deaths than influenza and RSV [42]. As these parameters are key drivers of cost-effectiveness outcomes, these factors directly contribute to the higher health gains and cost offsets achievable with COVID-19 vaccination, and consequently to the higher EJP. Differences in methodological choices across evaluations further contribute to the observed variation in EJPs between respiratory vaccines [9,40,41]. In most recent cost-effectiveness analyses of high-dose influenza vaccination, comparisons were typically made against standard-dose influenza vaccination, capturing only the incremental benefits of switching between vaccine types, rather than the full value of influenza vaccination versus no vaccination [40]. As a result, these analyses typically do not aim to assess the overall value of influenza vaccination programs when compared with evaluations assessing vaccination versus no vaccination, as this COVID-19 analysis has done. As such, the higher EJP estimated for COVID-19 vaccination appears consistent with both the underlying epidemiology and the analytical framework applied, and reflects the persistently higher disease burden of COVID-19 relative to other respiratory infections.

Previously discussed limitations [9,21,22] also apply to this evaluation. Uncertainty around the evolving incidence of COVID-19 was addressed by evaluating vaccination impact under low and high incidence scenarios; however, the true trajectory of COVID-19 incidence will only become clear over time. Our base case analysis was not intended to predict variant-driven resurgence waves, but rather to approximate the value of vaccination in a routine endemic setting. Analyses specifically addressing future resurgence would require alternative assumptions and potentially different modeling approaches that explicitly account for epidemic timing, variant characteristics, and changes in population susceptibility. The number of COVID-19 hospitalizations reported in the Netherlands (average of 2023 and 2024, 19,500 hospitalizations [8,44]) was comparable to the number predicted in the base case model (average of 2023/2024 and 2024/2025, ranging from 18,455 with mRNA1283 to 20,134 with BNT162b2), providing support for the base case incidence assumptions. A static model was used, which does not fully capture transmission dynamics, immunity effects, or indirect protection compared to dynamic transmission models. Fust et al. (2026) [21] found in a scenario analysis that incorporating indirect protection lowered ICER estimates. Therefore, the reported public health impact and cost-effectiveness of mRNA-1283 are likely conservative. Post-COVID-19 condition was not included in the base case analysis due to uncertainty in its current epidemiology and burden in the post-pandemic setting, as well as variability in its definition and measurement. However, scenario analyses including post-COVID-19 condition demonstrated additional health gains and economic value, indicating that the base case results may be considered conservative. The efficacy and safety of mRNA-1283 have not yet been established in the real-world setting. Thus, the rVE of mRNA-1283 versus mRNA-1273 from the NextCOVE trial [3], as well as the results of the indirect treatment comparison of mRNA-1283 and BNT162b2, should be validated and refined with real-world evidence studies. No data for the Netherlands (or Europe) were identified on utility loss associated with COVID-19; thus, the US data were used [9].

There remains a clear need to continue investing in COVID-19 vaccination in the Netherlands. Such programs are still highly relevant, given the ongoing burden of disease, and additionally, new COVID-19 vaccines such as the next-generation mRNA-1283 may provide an opportunity to optimize COVID-19 vaccination programs, especially for populations most vulnerable to severe COVID-19. Although overall incidence has declined since the peak of the pandemic, COVID-19 transmission remains unpredictable, with a persistent risk of renewed increases in incidence. In addition, while vaccination of all adults aged 50–59 years may be perceived as less critical from a public health perspective, it is highly relevant from a broader societal perspective, as preventing COVID-19 and other respiratory infectious diseases in this age group helps reduce productivity losses and work absenteeism, thus providing wider socioeconomic gains.

The findings of this analysis in the Netherlands may have broader relevance for other European settings. The value of COVID-19 vaccination is driven by key parameters, including incidence, risk of severe outcomes, vaccine effectiveness, healthcare costs, and vaccination coverage. Many European countries share similar demographics, such as aging populations, comparable healthcare systems, and seasonal respiratory infection patterns. As such, while absolute estimates of health impact and EJPs may differ across countries, the direction and magnitude of COVID-19 vaccination benefits are likely to be comparable. When interpreting findings in other settings, however, differences in country-specific factors, including healthcare costs, vaccination uptake, population risk profiles, and epidemiological dynamics, should be considered.

## 5. Conclusions

The findings support continued COVID-19 vaccination as a key public health measure to address the persistent health and societal burden associated with ongoing transmission of SARS-CoV-2. In this setting, optimizing vaccine choice within existing programs becomes increasingly important. The comparative analyses suggest that mRNA-1283 is potentially associated with the greatest health gains, based on evidence of potentially higher efficacy or effectiveness compared with originally licensed mRNA COVID-19 vaccines. Consequently, using mRNA-1283 could further enhance both health outcomes and economic benefits within COVID-19 vaccination programs.

## Figures and Tables

**Table 1:** Vaccine efficacy of mRNA-1283, mRNA-1273, and BNT162b2 [21].

Age Group (Years)	Comparison of mRNA-1273 & mRNA-1283			Comparison of mRNA-1283 & BNT162b2		
	2024/25 mRNA-1273 VE	rVE of mRNA-1283 vs mRNA-1273 (95% CI)	mRNA-1283 VE (Range)	mRNA-1283 VE	rVE of mRNA-1283 vs BNT162b2 (95% CI)	BNT162b2 VE (Range)
	Symptomatic Infection					
18–59 high-risk	48.2%	16.3% (1.8–28.7%)	56.6% (49.1–63.0%)	56.6%	19.0% (4.9–31.0%)	46.5% (37.2–54.4%)
≥60	54.5%	13.5% (−7.7–30.6%)	60.7% (50.8–68.4%)	60.7%	22.8% (3.7–38.1%)	49.0% (36.5–59.2%)
	Hospitalization ^†^					
18–59 high-risk	50.5%	38.1% (−6.6–64.1%)	69.4% (46.3–82.2%)	69.4%	44.1% (3.2–67.7%)	45.2% (5.1–68.3%)
≥60	57.2%	38.1% (−6.6–64.1%)	73.5% (53.3–84.6%)	73.5%	44.1% (3.2–67.7%)	52.6% (17.9–72.6%)

^†^ If VE against hospitalization is lower than VE against infection, the incremental protection against the probability of hospitalization given infection is set to 0. This assumption is aligned with Fust et al. 2026 [21], and is based on the observation that mRNA COVID-19 vaccines showed consistently higher protection against severe COVID-19 disease (i.e., hospitalization and death) than against COVID-19 infection. CI: confidence interval; rVE: relative vaccine effectiveness; VE: vaccine effectiveness; vs: versus.

**Table 2:** Impact of mRNA-1283 versus no vaccination.

	Base Case		High Incidence		Low Incidence	
	No Vaccine	mRNA-1283	No Vaccine	mRNA-1283	No Vaccine	mRNA-1283
Symptomatic infections	459,068	391,139	655,697	558,682	262,371	223,543
Averted	67,929		97,015		38,828	
Hospitalizations	23,830	18,455	34,037	26,359	13,620	10,548
Averted	5375		7677		3072	
Deaths	5348	4137	7638	5908	3057	2364
Averted	1211		1730		692	
Treatment costs (€)	346,486,776	279,941,386	494,893,571	399,850,768	198,027,466	159,992,774
Averted	66,545,390		95,042,961		38,034,692	
QALY loss	43,921	34,254	62,732	48,890	25,103	19,613
QALY gain	9667		13,842		5490	
EJP (WTP €50k)	€237.93		€349.47		€126.34	

EJP: economically justifiable price; k: thousand; QALY: quality-adjusted life-year; WTP: willingness-to-pay.

**Table 3:** Additional scenario analyses of mRNA-1283 versus no vaccination.

	Hospitalizations Averted	Deaths Averted	EJP (WTP €50,000/QALY)
Base case	5375	1211	237.93
VE infection and hospitalization 95% CI lower bound	3754	846	160.70
VE infection and hospitalization 95% CI upper bound	6693	1508	301.05
rVE hospitalization = rVE infection	4532	1021	199.27
VCR − 20%	4300	969	237.93
VCR + 20%	6451	1453	237.93
Expand to all 50–59-year-olds	5481	1219	186.54
≥75 years only	2779	628	371.03
Post-COVID-19 condition	Additional 2265 post-COVID-19 condition cases prevented		241.96

EJP: economically justifiable price; QALY: quality-adjusted life-year; WTP: willingness-to-pay.

**Table 4:** Impact of mRNA-1283 versus mRNA-1273.

	Base c.		High Incidence		Low Incidence		Lower Bound rVE		Upper Bound rVE	
	mRNA-1273	mRNA-1283	mRNA-1273	mRNA-1283	mRNA-1273	mRNA-1283	mRNA-1273	mRNA-1283	mRNA-1273	mRNA-1283
Symptomatic infections	400,070	391,139	571,437	558,682	228,648	223,543	400,070	405,036	400,070	380,290
Averted	8931		12,755		5105		−4966		19,780	
Hospitalizations	19,764	18,455	28,230	26,359	11,296	10,548	19,764	20,077	19,764	17,563
Averted	1309		1871		748		−312		2201	
Deaths	4432	4137	6330	5908	2533	2364	4432	4502	4432	3936
Averted	295		422		169		−70		496	
Treatment costs	294,856,989	280,113,088	421,155,221	400,096,059	168,517,334	160,090,990	294,856,989	298,821,108	294,856,989	269,157,438
Averted	14,743,901		21,059,162		8,426,344		−3,964,119		25,699,552	
QALY loss	36,571	34,248	52,206	45,787	20,930	19,609	36,571	37,159	36,571	32,633
QALY gain	2323		3325		1321		−588		3938	
Incremental EJP (WTP 50k)	€61.53		€88.06		€35.00		NA		€104.65	
Incremental EJP (WTP 20k)	€28.77		€41.16		€16.37		NA		€49.11	

EJP: economically justifiable price; k: thousand; NA: indicates negative incremental EJP of mRNA-1283 over mRNA-1273; QALY: quality-adjusted life-year; rVE: relative vaccine efficacy; WTP: willingness-to-pay.

**Table 5:** Impact of mRNA-1283 versus BNT162b2.

	Base Case		High Incidence		Low Incidence		Lower Bound rVE		Upper Bound rVE	
	BNT162b2	mRNA-1283	BNT162b2	mRNA-1283	BNT162b2	mRNA-1283	BNT162b2	mRNA-1283	BNT162b2	mRNA-1283
Symptomatic infections	407,637	391,139	582,245	558,682	232,973	223,543	393,312	391,139	424,954	391,139
Averted	16,499		23,563		9430		2173		33,815	
Hospitalizations	20,134	18,455	28,757	26,359	11,507	10,548	18,527	18,455	21,424	18,455
Averted	1679		2398		959		72		2969	
Deaths	4515	4137	6448	5908	2580	2364	4153	4137	4806	4137
Averted	378		540		216		16		669	
Treatment costs	299,940,303	280,113,232	428,415,830	400,096,059	171,422,591	160,090,990	281,291,036	280,113,232	316,075,383	280,113,232
Averted	€19,827,071		€28,319,771		€11,331,602		€1,177,805		€35,962,152	
QALY loss	37,253	34,248	53,181	48,881	21,319	19,609	34,368	34,248	39,598	34,248
QALY gain	3005		4300		1710		120		5350	
Incremental EJP (WTP 50k)	€79.96		€114.38		€45.52		€3.37		€142.67	
Incremental EJP (WTP 20k)	€37.57		€53.74		€21.40		€1.68		€67.21	

EJP: economically justifiable price; k: thousand; QALY: quality-adjusted life-year; rVE: relative.

## Data Availability

The data presented in this study are available in this article.

## Abbreviations

The following abbreviations are used in this manuscript:

BNT162b2	Comirnaty, Pfizer-BioNTech
CI	Confidence interval
DSA	Deterministic sensitivity analysis
EJP	Economically justifiable price
ICER	Incremental cost-effectiveness ratio
ICU	Intensive care unit
K	thousands
M	million
mRNA-1273	Spikevax, Moderna
mRNA-1283	mNEXSPIKE, Moderna
NNV	Number needed to vaccinate
PSA	Probabilistic sensitivity analysis
QALY	Quality-adjusted life-year
rVE	Relative vaccine effectiveness
US	United States
VCR	Vaccine coverage rate
VE	Vaccine effectiveness
WTP	Willingness-to-pay

## Appendix A

**Table A1:** Model inputs.

Population					Source
Total	5,922,720		High-risk		[36]
60+	4,784,093		Age-based		
50–59	298,007		Yes		
18–49	840,620		Yes		
Incidence COVID-19 symptomatic infection					
Age- and month-dependent					Table A2 [9]
Proportion of infected people seeking care at the general practitioner level					
60+			28.46%		Age-adjusted [38]
50 to 59			21.26%		
18 to 49			19.69%		
Age-dependent COVID-19 hospitalization risk given COVID-19 symptomatic infection					
60+			5.89%		Age-adjusted [9]
50 to 59			0.68%		
18 to 49			0.26%		
Percentage Intensive Care Unit (ICU) among hospitalized					
60+			5.79%		Age-adjusted [9]
50 to 59			11.72%		
18 to 49			11.43%		
In-hospital mortality (no ICU)					
60+			18.04%		Age-adjusted [9]
50 to 59			2.10%		
18 to 49			0.60%		
In-hospital mortality (ICU)					
60+			43.68%		Age-adjusted [9]
50 to 59			15.05%		
18 to 49			6.74%		
Hospital readmission rate			11.70%		[45,46]
Post-discharge mortality			3.80%		[46]
Infection associated myocarditis					
	Female		Male		
60+	0.0847%		0.1683%		Age-adjusted [9]
50 to 59	0.1018%		0.1881%		
18 to 49	0.0592%		0.1682%		
Vaccine characteristics					
Vaccine effectiveness (VE)			Age- and product-dependent		Table 1 (main manuscript)
Waning of VE (same for all products)					
Against infection			4.75%		[32]
Against hospitalization			2.46%		[33]
Vaccine related AEs					
Probabilities		mRNA-1283	mRNA-1273	BNT162b2	
Grade 3 local		1.61%	1.17%	1.17%	[3]
Grade 3 systemic		7.16%	5.77%	5.77%	
Anaphylaxis		0.0005%	0.0005%	0.0005%	[47]
Myocarditis/pericarditis *		0.0008%	0.0008%	0.0008%	[34]
Vaccination coverage					
	Sep-25	Oct-25	Nov-25	Dec-25	[10]
60+	1.90%	19.37%	35.63%	41.60%	
50 to 59	11.68%	18.51%	23.55%	26.93%	
18 to 49	2.93%	4.65%	5.91%	6.76%	
Costs (Euro)					
Vaccine administration cost			13.67		[48] inflated to 2025
Direct healthcare cost					
Outpatient care (GP visit)			43.22		[49] inflated to 2025
Hospital General Ward (same for readmission)			7051.88		[50] inflated to 2025
Hospitalized—ICU (same for readmission)			12,650.72		
Hospitalization recovery (inpatient follow-up)			119.20		[49] inflated to 2025
Post-infection period (long-COVID) (scenario)			1206.29		[51] inflated to 2025
Myocarditis following infection			3304.61		Age-adjusted [9] inflated to 2025
AEs					
Grade 3 local			0.26		[52] inflated to 2025
Grade 3 systemic			0.26		
Anaphylaxis			339.26 (1 ED visit)		[49] inflated to 2025
Myocarditis/pericarditis (5.3 nursing days)			6119.47		[42,49] inflated to 2025
Indirect costs					
Percentage in labor force					
60+			22.5%		[9]
50 to 59			83.0%		
18 to 49			85.9%		
Daily wage			208.74		[49,53] inflated to 2025
Productivity loss estimates (in days)					
Vaccine administration			0.13		1 h assumption
COVID-19 non-hospitalized			3.5		[54]
COVID-19 hospitalized (non ICU)			8.43 (SD 7.51)		[39]
COVID-19 hospitalized (ICU)			16.64 (SD 13.69)		[39]
Recovery from hospitalization			27		[55]
Post-COVID (scenario)			11		[56]
Myocarditis (infection-induced)			8.8		Age-adjusted [9]
Myocarditis (adverse events)			3		[42]
Anaphylaxis (adverse event)			1		[42]
QALY loss					
Baseline utility data for each population group: age-dependent					
Grade 3 local			0.0004		[57]
Grade 3 systemic			0.0004		
Anaphylaxis			0.02		
Myocarditis/pericarditis			0.0019		
COVID-19 Infection related health states (>18 years)					
No formal care			0.0046		Assumed based on [58] (assumed same for readmission)
Outpatient care			0.0046		
Hospitalized/readmission					
No ICU			0.0174		
ICU only			0.0394		
Ventilator			0.0394		
Myocarditis (infection)			0.0188		Age-adjusted [9]
Post-COVID (scenario)					
No formal care			0.0		[59] adjusted for outpatient care QALY loss [60]
Outpatient care			0.023		
Hospitalization			0.1026		

* Myocarditis/pericarditis applies to ages 18–60 only; AE rates for BNT162b2 assumed same as mRNA-1273. No Grade 4 event assumed for mRNA-1273 and BNT162b2.

**Table A2:** COVID-19 symptomatic infections incidence (%) stratified by age and month.

Age Group	Sep-25	Oct -25	Nov-25	Dec-25	Jan -26	Feb -26	Mar -26	Apr -26	May-26	Jun -26	Jul -26	Aug-26
18–49 years	0.10	1.23	0.57	0.82	0.49	0.76	0.65	0.25	0.12	0.05	0.04	0.10
50–59 years	0.06	1.23	0.52	0.81	0.57	0.55	0.64	0.23	0.13	0.05	0.05	0.06
60–100 years	0.10	2.53	0.81	1.10	0.85	0.92	1.16	0.46	0.23	0.06	0.07	0.10

**Table A3:** Probabilistic sensitivity analyses parameters used for comparisons of mRNA-1283 versus mRNA-1273 and mRNA-1283 versus BNT162b2.

Variable	Age Group	Point Estimate	Distribution	SE	Details
COVID incidence rates	All age groups	Age- and month-specific	Normal	10%	Multiplier applied to all incidence rates to vary them all up or down for a certain percentage. A multiplier of 1.0 represents the base case values. 0.10 or 10% of the multiplier was used as the SE for the multiplier to vary incidence rates in the PSA.
Vaccine Waning (intervention): Infections	18–100 years	0.048	N/A	N/A	Set equal to vaccine waning for comparator. Varies as the waning of the comparator varies following [21]
Vaccine Waning (intervention): Hospitalizations	18–100 years	0.025	N/A	N/A	Set equal to vaccine waning for comparator. Varies as the waning of the comparator varies following [21]
Hospitalization rates	18–49 years	0.255%	Beta	0.0002552	SE estimated as 10% of the mean.
	50–59 years	0.68%	Beta	0.00068	
	60–100 years	5.89%	Beta	0.0059	
Proportion No formal care	NFC: 18–49 years	80.0%	Beta	0.0800	SE estimated as 10% of the mean.
	NFC: 50–59 years	78.1%	Beta	0.0781	
	NFC: 60–100 years	64.5%	Beta	0.0645	
Proportion Outpatient care	OP: 18–49 years	19.7%	N/A		Calculated as 1- Hospitalization rates—proportion outpatient care.
	OP: 50–64 years	21.3%	N/A		
	OP: 65–100 years	29.6%	N/A		
In-Hospital Level of Care: No ICU or Ventilator, ICU including ICU only and ICU with Ventilator	ICU: 18–49 years	11.3%	Dirichlet		Alpha = mean × 100 beta = 1 Gamma distribution used to select values for each of the 3 inputs across each age group and then converted to probabilities. Inputs for each age group add to 100%.
	ICU: 50–59 years	11.72%	Dirichlet		
	ICU: 60–100 years	5.79%	Dirichlet		
	No ICU or Vent: 18–49 years	88.57%	Dirichlet		
	No ICU or Vent: 50–59 years	88.28%	Dirichlet		
	No ICU or Vent: 60–100 years	94.21%	Dirichlet		
Probability Readmission	All age groups and level of care	11.7%	Beta	0.0117	SE estimated as 10% of the mean
In-hospital mortality: ICU including ICU only and ICU with ventilator	18–49 years	0.067	Dirichlet		alpha = mean × 100 beta = 1 Gamma distribution used to select values for each of the 2 inputs (in hospital mortality and no readmission) across an age group and level of care, then converted to probabilities in conjunction with the rate of readmission to add to 100%. These two probabilities will combine to represent 100%—% readmission.
	50–59 years	0.151	Dirichlet		
	60–100 years	0.437	Dirichlet		
No readmission or mortality: ICU including ICU only and ICU with ventilator	18–49 years	0.816	Dirichlet		
	50–59 years	0.733	Dirichlet		
	60–100 years	0.446	Dirichlet		
In-hospital mortality: No ICU or ventilator	18–49 years	0.006	Dirichlet		
	50–59 years	0.021	Dirichlet		
	60–100 years	0.18	Dirichlet		
No readmission or mortality: No ICU or ventilator	18–49 years	0.877	Dirichlet		
	50–59 years	0.862	Dirichlet		
	60–100 years	0.703	Dirichlet		
Post-discharge mortality	All age groups and levels of care	3.80%	Beta	0.0039%	SE estimated from the upper and lower bounds of the 95%CI (approximated +/−20% of base case) and assuming an approximate normal distribution.
Vaccine administration cost	All age groups	€13.67	Gamma	€1.367	SE estimated as 10% of the mean.
AE related costs	All age groups	€0.26	Gamma	€0.026	SE estimated as 10% of the mean.
Grade 3 local		€0.26		€0.026	
Grade 3 systemic		€339.30		€33.93	
Anaphylaxis		€3304.6		€330.46	
Myocarditis/Pericarditis		0			
COVID-19 caused myocarditis	All age groups	€6119.5	Gamma	€611.95	SE estimated as 10% of the mean.
Outpatient care costs	All age group: physician costs	€43.20	Gamma	€4.32	SE estimated as 10% of the mean.
Hospitalization costs	All age groups: No ICU or Vent	€7052	Gamma	€705.20	SE estimated as 10% of the mean.
	All age groups: ICU including ICU only and ICU with ventilator	€12,650.70	Gamma	€1260.51	
Time loss Adverse events (days)	All ages: Any Grade 3 local adverse events	0	N/A	0	N/A SE estimated as 10% of the mean.
	All ages: Any Grade 3 systemic adverse events	0	N/A	0	
	All ages: Anaphylaxis	1	Gamma	0.1	
	All ages: Myocarditis/Pericarditis	3	Gamma	0.3	
Time loss for Acute COVID phase (days)	All ages: Outpatient and not seeking care	3.5	Gamma	0.035	SE estimated as 10% of the mean.
	All ages: Symptoms prior to hospitalization	3.5	Gamma	0.035	
	All ages: In hospital stay	8.43 (no ICU) 16.64 (ICU)	Gamma	0.0843 (no ICU) 1.664 (ICU)	
	All ages: Recovery	27.0	Gamma	2.7	
	All ages: Readmission	8.43 (no ICU) 16.64 (ICU)	Gamma	0.843 (no ICU) 1.664 (ICU)	
Time loss due to COVID-19 caused myocarditis	All age groups	8.82 days	Gamma	0.882	SE estimated as 10% of the mean.
Daily wage	€208.7	All ages	Gamma	€20.87	
QALY loss Adverse events	All ages: Any Grade 3 local adverse events	0.0004	Beta	0.00004	SE estimated as 10% of the mean.
	All ages: Any Grade 4 local adverse events	0.0019	Beta	0.00019	
	All ages: Any Grade 3 systemic adverse events	0.0004	Beta	0.00004	
	All ages: Any Grade 4 systemic adverse events	0.0019	Beta	0.00019	
	All ages: Anaphylaxis	0.0019	Beta	0.00019	
	All ages: Myocarditis/Pericarditis	0.0006	Beta	0.00006	
QALY lossCOVID-19 associated myocarditis	All ages	0.02	Beta	0.0002	SE estimated as 10% of the mean.
Non hospitalized patients QALY (18+)	18+: No formal care	0.0046	Beta	0.00046	SE estimated as 10% of the mean.
	18+: Outpatient care	0.0046	Beta	0.00046	
Hospitalization QALY losses (18+)	18+: No ICU or Vent (Symptoms prior to hosp)	0.000	Beta	0.000	SE estimated as 10% of the mean.
	18+: ICU only (Symptoms prior to hosp)	0.000	Beta	0.000	
	18+: Vent (Symptoms prior to hosp)	0.000	Beta	0.000	
	18+: No ICU or Vent (In-hospital)	0.017	Beta	0.031	
	18+: ICU only (In hospital)	0.039	Beta	0.058	
	18+: Vent (In hospital)	0.039	Beta	0.058	
Readmission QALY losses (18+)	18+: No ICU or Vent	0.0174	Beta	0.00174	SE estimated as 10% of the mean.
	18+: ICU only	0.0394	Beta	0.00394	
	18+: Vent	0.0394	Beta	0.00394	

CI: confidence interval; hosp: hospitalization; ICU: intensive care unit; N/A, not applicable; QALY: quality-adjusted life-year; SE: standard error.

**Table A4:** Probabilistic sensitivity analyses parameters for vaccine effectiveness and vaccine acquisition cost for mRNA-1283 versus mRNA-1273 and mRNA-1283 versus BNT162b2.

Scenario: mRNA-1273 Versus mRNA-1283					
rVE: Infections	18–49 years	0.163	Beta	0.0686	SE estimated from the upper and lower bounds of the 95%CI (including negative estimates for LB) and assuming an approximate normal distribution.
	50–59 years	0.163	Beta	0.0686	
	60–100 years	0.135	Beta	0.0977	
rVE: Hospitalizations	18–49 years	0.381	Beta	0.1804	SE estimated from the upper and lower bounds of the 95%CI (including negative estimates for LB) and assuming an approximate normal distribution.
	50–59 years	0.381	Beta	0.1804	
	60–100 years	0.381	Beta	0.1804	
1283 Vaccine Effectiveness: Infections	18–49 years	0.566	N/A	N/A	Calculated based on comparator (i.e., mRNA-1273) VE and rVE selection. Varies as other values vary. Maximum value of 0.99 included in calculations.
	50–59 years	0.566			
	60–100 years	0.607			
1283 Vaccine Effectiveness: Hospitalizations	18–49 years	0.694	N/A	N/A	Calculated based on comparator (i.e., mRNA-1273) VE and rVE selection. Varies as other values vary. Maximum value of 0.99 included in calculations.
	50–59 years	0.694			
	60–100 years	0.735			
Scenario: mRNA-1283 versus BNT162b2					
rVE: Infections	18–49 years	0.190	Beta	0.0190	SE estimated as 10% of the mean.
	50–59 years	0.190	Beta	0.0190	
	60–100 years	0.228	Beta	0.0228	
rVE: Hospitalizations	18–49 years	0.441	Beta	0.0441	SE estimated as 10% of the mean.
	50–59 years	0.441	Beta	0.0441	
	60–100 years	0.441	Beta	0.0441	
BNT162b2 Vaccine Effectiveness: Infections	18–49 years	0.465	N/A	N/A	Calculated based on comparator (i.e., mRNA-1283) VE and rVE selection. Varies as other values vary. Maximum value of 0.99 included in calculations.
	50–59 years	0.465			
	60–100 years	0.490			
BNT162b2 Vaccine Effectiveness: Hospitalizations	18–49 years	0.452	N/A	N/A	Calculated based on comparator (i.e., mRNA-1283) VE and rVE selection. Varies as other values vary. Maximum value of 0.99 included in calculations.
	50–59 years	0.452			
	60–100 years	0.526			

CI: Confidence interval; N/A, Not applicable; SE: Standard error.
